# Supplementary material for: The Role of Complementary Feeding Practices in Addressing the Double Burden of Malnutrition among Children Aged 6–23 Months: Insight from the Vietnamese General Nutrition Survey 2020
Source: Nutrients. 2024 Sep 25;16(19):3240. doi: 10.3390/nu16193240 (PMC11478371; doi:10.3390/nu16193240)
Supplement: Supplementary file 1 [file nutrients-16-03240-s001.zip › nutrients-3192398-supplementary.pdf]

Supplementary Materials

The role of complementary feeding practices in addressing the double burden of malnutrition among children aged 6-23 months: Insight from the Vietnamese General Nutrition Survey 2020

Pui Yee Tan [1], Somphos Vicheth Som [1,2], Son Duy Nguyen [3,4], Do Tranh Tran [4], Nga Thuy Tran [5], Van Khanh Tran [5], Louise Dye [1,6], Bernadette J. Moore [1], Samantha Caton [7], Hannah Ensaff [1], Xiaodong Lin [8], Geoffry Smith [9], Pauline Chan [9] and Yun Yun Gong [1,*]

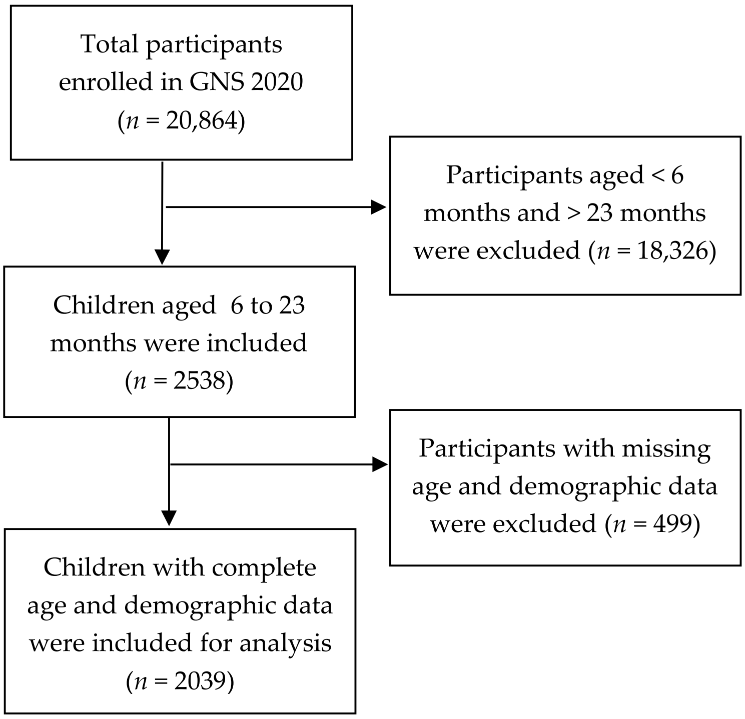

Supplementary Figure S1: Flow diagram of data selection from the General Nutrition Survey (GNS) 2020

Supplementary Table S1. Numbers and proportions of missing data for each variable

Variable	Number of observations	Missing data	
		Number	%
**Demographic and socioeconomic indicators**			
Age	2039	0	0
Sex	2039	0	0
Area of residence	2039	0	0
Geographical area	2039	0	0
Ethnicity	2039	0	0
Wealth index	2039	0	0
**Nutritional status**			
Stunting	1734	305	15·0
Underweight	1739	300	14·7
Wasting	1733	306	15·0
Overweight	1733	306	15·0
**Micronutrient deficiencies**			
Anaemia (hemoglobin)	1822	217	10·6
Iron deficiency (serum ferritin)	1728	311	15·3
Low serum retinol (serum retinol)	1784	255	12·5
Low serum zinc (serum zinc)	1820	219	10·7
Inflammation (CRP and AGP)	1747	292	14·3
**IYCF practice indicators**			
Ever breastfed	2028	11	0·5
Early initiation of breastfeeding	2028	11	0·5
Continued breastfeeding at 12-23 months*	1308	0	0
Introduction of solid, semi-solid, or soft foods 6–8 months**	344	0	0
Minimum dietary diversity	2028	11	0·5
Minimum meal frequency	2028	11	0·5
Minimum milk feeding frequency***	793	793	0
Minimum acceptable diet	2028	11	0·5
Animal-source foods consumption	2028	11	0·5
Sweetened beverages consumption	2028	11	0·5
Unhealthy food consumption	2028	11	0·5
Zero vegetable or fruit consumption	2028	11	0·5

AGP, α-1-acid glycoprotein; CRP, c-reactive protein; IYCF, infant and young children feeding.

*only applicable to infants aged 12-23 months.

**only applicable to infants aged 6-8 months.

***only applicable to non-breastfed infants

Supplementary Table S2. Logistic regressions between food group consumption and demographic and socioeconomic determinants among Vietnamese children

Variables		Breast milk	Grains, roots and tubers	Legumes, nuts, and seeds	Dairy products	Flesh foods	Eggs	Vitamin A rich F&V	Other F&V
		**OR (95% CI)**	**OR (95% CI)**	**OR (95% CI)**	**OR (95% CI)**	**OR (95% CI)**	**OR (95% CI)**	**OR (95% CI)**	**OR (95% CI)**
Sex (Ref: females)	Males				1·23 (1·05, 1·45) *				
Age (Ref: 6-11 months)	12-23 months		2·29 (1·11, 4·76) *	1·96 (1·14, 3·38) *	3·30 (2·56, 4·25) ***	1·98 (1·49, 2·64) ***	1·54 (1·02, 2·31) *		1·53 (1·21, 1·94) ***
Area of residence (Ref: urban)	Rural				0·96 (0·68, 1·34)	1·10 (0·72, 1·71)	0·67 (0·46, 0·98) *	0·70 (0·54, 0·91) *	0·74 (0·51, 1·07)
	Mountainous	0·37 (0·17, 0·80) *			0·65 (0·43, 0·99) *	0·39 (0·21, 0·72) **	1·37 (0·86, 2·18)	0·57 (0·30, 1·10)	0·56 (0·36, 0·85) **
Wealth quintiles (Ref: poorest)	Poorer	2·05 (1·04, 4·04) *				1·71 (1·03, 2·84) *		1·34 (0·72, 2·47)	
	Middle	2·35 (1·06, 5·25) *				3·13 (2·02, 4·81) ***		1·73 (1·10, 2·75) *	
	Richer	3·86 (1·71, 8·73) **				3·34 (1·70, 6·55) ***		2·42 (1·43, 4·09) **	
	Richest	3·53 (1·68, 7·42) **				3·77 (2·19, 6·48) ***		2·94 (1·73, 5·01) ***	
Ethnicity (Ref: Kinh major)	Minorities	0·31 (0·17, 0·56) ***			0·62 (0·42, 0·92) *	0·25 (0·18, 0·34) ***	1·84 (1·22, 2·76) **	0·48 (0·32, 0·70) ***	
Geographical area (Ref: Northern mountains)	Red River Delta	2·80 (1·08, 7·24) *			1·48 (0·97, 2·27)	2·53 (0·98, 6·52)	0·59 (0·36, 0·95) *		1·61 (1·03, 2·54) *
	North Central and Central Coastal	1·45 (0·55, 3·79)			1·16 (0·60, 2·26)	2·51 (1·22, 5·15) *	0·45, (0·31, 0·66) ***		1·15 (0·70, 1·90)
	Central Highlands	0·90 (0·18, 4·41)			0·91 (0·34, 2·44)	0·71 (0·24, 2·06)	0·69 (0·32, 1·49)		1·24 (0·44, 3·51)
	Southeast	3·26 (1·32, 8·08) *			2·34 (1·71, 3·20) ***	2·60 (1·21, 5·58) *	0·65 (0·36, 1·19)		1·33 (0·69, 2·58)
	Mekong River Delta	2·59 (1·08, 6·20) *			1·12 (0·55, 2·29)	1·64 (0·72, 3·76)	0·18 (0·10, 0·34) ***		1·60 (0·91, 2·82)

Bivariate logistic regressions were performed and OR with its respective 95% CI were reported.
* p< 0·05; ** p< 0·01; *** p< 0·001.
CI, confidence intervals; F&V, fruits and vegetables; OR, odds ratio; Ref, reference group.
